# Infection prevention and control programme priorities for sustainable health and environmental systems

**DOI:** 10.1186/s44263-023-00031-4

**Published:** 2024-02-01

**Authors:** Gemma L. Saravanos, Md Saiful Islam, Yuanfei Huang, Jocelyne M. Basseal, Holly Seale, Brett G. Mitchell, Meru Sheel

**Affiliations:** 1 Sydney Infectious Diseases Institute, Faculty of Medicine and Health, University of Sydney, Camperdown, NSW Australia; 2 Susan Wakil School of Nursing and Midwifery, Faculty of Medicine and Health, University of Sydney, Camperdown, NSW Australia; 3 School of Population Health, Faculty of Medicine and Health, University of New South Wales, Randwick, NSW Australia; 4 National Centre for Immunisation Research & Surveillance, Westmead, NSW Australia; 5 Sydney Medical School, Faculty of Medicine and Health, University of Sydney, Camperdown, NSW Australia; 6 School of Nursing, Avondale University, Central Coast Local Health District, Gosford, NSW Australia; 7 School of Nursing and Midwifery, Monash University, Victoria, Australia; 8 Sydney School of Public Health, Faculty of Medicine and Health, University of Sydney, Camperdown, NSW Australia

Infection prevention and control (IPC) programmes reduce infection risk for patients, health workers, and the community. They are fundamental to achieving resilient, responsive, and sustainable health systems that align with the Sustainable Development Goals. Paradoxically, IPC programmes contribute to environmental harm, and this must be addressed alongside longstanding programme priorities.

## Background

The discipline of infection prevention and control (IPC) lies at the intersection of clinical practice and public health, It encompasses a broad range of practices which aim to reduce the risk of infection for patients, health workers, and the wider community, and combat the spread of antimicrobial resistance (AMR) [1]. IPC policies and practices, henceforth ‘IPC programmes’, span all areas of the health system and sit at the core of healthcare safety and quality, global health security, and health emergency response [1]. Further, IPC programmes are fundamental to meeting the United Nations Sustainable Development Goals (SDGs), and this is strongly reflected in the inaugural World Health Organization (WHO) Global Strategy of IPC [2]. Paradoxically, IPC programmes can be resource-intensive, and the environmental impacts of this are in the early stages of being recognised and characterised [3, 4].

In this comment, we present a case for increased and ongoing investment in IPC programmes as essential for sustainable health and environmental systems. First, we illustrate the intersections between IPC programmes, health system sustainability and the SDGs. We then consider the current evidence base of IPC programmes, provide an overview of their environmental impacts, and explore some behavioural aspects of IPC programme implementation. We highlight three key priority areas for investment in IPC programmes needed to support sustainable health and environmental systems, and to advance the aims of the SDGs.

## IPC programmes and health system sustainability

**Fig. 1:**
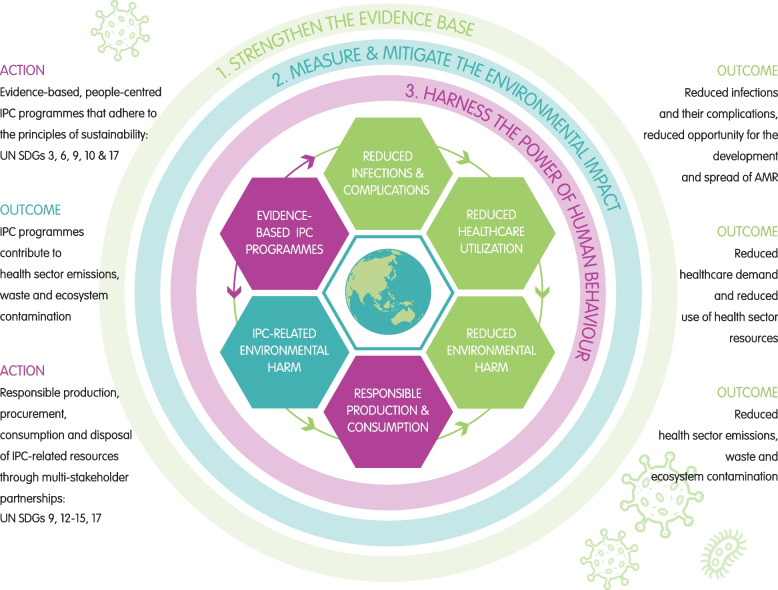
A conceptual framework of relationships and priorities in infection prevention and control for sustainable health and environmental systems. Legend: We highlight three key IPC programme priorities for sustainable health and environmental systems, and these are **1** Strengthen the evidence base—invest in high-quality mixed-method research and surveillance to evaluate IPC programmes. **2** Measure and mitigate environmental impacts—invest in systems and research to measure and mitigate the environmental impacts of IPC practices. **3** Harness the power of human behaviour—invest in the health workforce to create an enabling environment for IPC programmes that are evidence-based, people-centred, and adhere to the principles of sustainability. Acronyms: AMR, antimicrobial resistance; IPC, infection prevention and control; SDGs, sustainable development goals; UN, United Nations; water sanitation and hygiene (WASH)

Infectious diseases impose a substantial and inequitable societal burden, and addressing this is a clear global health priority [2, 5]. Robust IPC programmes are integral to responding to global infectious disease challenges and achieving resilient, responsive, and sustainable health systems that align with the SDGs, reduce health costs, and deliver safer health care for all (Fig. 1) [2, 5]. The aims of IPC programmes are well aligned with those of sustainable health systems; they prioritise health promotion and disease prevention and this carries the co-benefit of reducing a variety of downstream economic, social and environmental impacts [6].

Existing IPC programme guidelines focus on reducing infections and AMR in health facilities. However, there is increasing recognition of the benefits of tailored programmes in primary care settings and improved integration within the broader community [2]. This has been starkly highlighted during epidemics of new and high-consequence infectious diseases such as COVID-19 and Ebola, where community-level IPC practices have been crucial to control disease spread and mitigate societal impact [2, 7]. Strengthening partnerships with communities and with other health programmes such as immunisation, public health and emergency response, can support improved IPC preparedness and response beyond health facilities [2].

## IPC programmes are evidence-based

A recognised approach to improving the sustainability of health systems is to identify and minimise ‘low value’ care, that is, activities that do not have sound evidence of improving health outcomes [6]. While the principles of IPC are well established and there is good evidence that IPC programmes are cost-effective, the majority of evidence is derived from low-quality studies. Robust effectiveness and cost-effectiveness studies have mostly been undertaken in high-income countries and only evaluate IPC interventions for a narrow range of infections and outcomes [2].

IPC interventions with a low certainty of evidence for improving health outcomes should be critically evaluated, particularly those associated with substantial economic, social, and environmental costs. One key example is the widespread use of single-use gowns and gloves to prevent multi-drug resistant organism transmission. This practice is broadly recommended in IPC guidelines, however for the common AMR pathogens *multi-resistant Staphylococcus aureus* and *vancomycin-resistant enterococci*, an accumulating body of observational evidence suggests that this practice provides no additional benefit over hand hygiene and environmental cleaning [8]. Notably, the environmental costs of this practice may be substantial, making it a priority for evaluation [3].

There is a call for dedicated funding streams and capacity-building for IPC research to support the evaluation of the effectiveness and impacts of IPC programmes in all settings, as well as the consequences of inaction [2]. Traditional randomised controlled trials can be useful for answering focused research questions [8]; however, this approach alone cannot adequately address complex IPC challenges. High-quality, mixed-method approaches are needed to identify and implement effective IPC interventions in complex systems [2]. Embedding research into existing IPC operations, such as surveillance, can support the resilience of research capacity over time and should be prioritised [2].

## IPC programmes have an environmental impact

The Lancet Countdown report on health and climate change estimated that emissions from the health sector contributed 4.6% of all global green house gas emissions in 2020 [9]. The production, procurement, consumption, and disposal of IPC-related resources is an important contributor to emissions, and this is increasingly being recognised and quantified in the literature [3]. Contamination of natural ecosystems with IPC-related waste and microplastics and clinically significant pathogens is also an important concern [4]. The COVID-19 pandemic increased the demand for single-use personal protective equipment (PPE), hand sanitiser, diagnostic tests, and vaccines, all of which collectively challenged supply chains, exceeded waste management capacities, and contaminated natural ecosystems [3, 4].

There is an urgent need to measure and mitigate the environmental impacts of IPC programmes. A life-cycle assessment, which considers the carbon footprint of a product from manufacture to disposal, can support an understanding of the environmental impacts of IPC programmes and how these can be mitigated [3, 4]. An analysis of PPE used in the United Kingdom during the first 6 months of the COVID-19 pandemic estimated that over 100,000 tonnes of carbon dioxide equivalent were generated [3]. Additionally, it was estimated that a reduction of up to 75% could be achieved through feasible mitigation strategies [3]

Practical approaches to mitigation include promoting rational, evidence-based use of IPC resources, and optimising processes related to the manufacture, procurement, disposal, reuse, and recycling of IPC-related products [3, 4]. Importantly, there is complexity in ensuring that the safety and effectiveness of IPC practises are maintained, and environmental outcomes improved. Innovative solutions must be co-developed through multi-stakeholder partnerships with expertise in IPC, industry, and environmental science [4]. These efforts will contribute to improving the sustainability and efficiency of IPC programmes while advancing the SDGs related to responsible production and consumption of resources, infrastructure and innovation, climate action, preservation of natural ecosystems and partnership for the goals [5].

## Evidence-based, sustainable IPC programmes are people-centred

Amongst all this, it is critical to acknowledge that IPC goes beyond being a technical discipline and that the success of a programme depends on the behaviours of the people within the system. A social science approach that centres on the health community is crucial to achieving an enabling environment for IPC programmes that are both evidence-based and adhere to the principles of sustainability [2, 7]. A myriad of contextual barriers must be identified and addressed. Resource scarcity and access to water, sanitation and hygiene (WASH), remain a persistent challenge to meeting the fundamentals of IPC in many health facilities located in low-income countries [1]. Suboptimal infrastructure for waste management, re-processing, and recycling is widespread and limits the opportunity for sustainable practices [1, 3, 4]. Non-evidence-based practices arise from a complex interplay of social, professional, and emotional factors; key examples include the overuse and misuse of resources such as antibiotics and non-sterile clinical gloves [1, 10].

Realising the full potential of IPC programmes will require increased and ongoing investment to ensure an educated, motivated, and supported health workforce who will drive behaviours consistent with a culture of evidence-based and sustainable IPC. Urgent priorities include ensuring equitable access to IPC resources and WASH; optimising the production, procurement, use and disposal of IPC-related resources; improving infrastructure for waste management and reprocessing of re-usable items; and embedding sustainability principles into existing IPC, education and training, and quality frameworks [1–3, 5, 6]. These actions must be underpinned by effective governance and leadership at all levels of the health system, alongside strong multi-stakeholder partnerships [2]. The WHO Global Strategy of IPC is well placed to drive this agenda but must be supported by political will that sets ambitious targets and upholds the accountability of key stakeholders [2].

## Conclusions

Robust IPC programmes are integral to achieving resilient, responsive, and sustainable health systems that align with the SDGs, reduce health costs, and deliver safer health care for all. Paradoxically, IPC programmes are an important contributor to health sector emissions, waste, and ecosystem contamination. Increased and equitable investment is needed to innovate and evaluate IPC programmes with regard to key health and environmental outcomes in all settings. This must be underpinned by effective governance and leadership, strong multi-stakeholder partnerships, and health community activation.

## Data Availability

Not applicable.
